# Early Evidence of Shifts in Alpine Summit Vegetation: A Case Study From Kashmir Himalaya

**DOI:** 10.3389/fpls.2020.00421

**Published:** 2020-04-24

**Authors:** Maroof Hamid, Anzar Ahmad Khuroo, Akhtar Hussain Malik, Rameez Ahmad, Chandra Prakash Singh, Jiri Dolezal, Shiekh Marifatul Haq

**Affiliations:** ^1^Centre for Biodiversity & Taxonomy, Department of Botany, University of Kashmir, Srinagar, India; ^2^Space Applications Centre, Indian Space Research Organization, Ahmedabad, India; ^3^Institute of Botany, The Czech Academy of Sciences, Pruhonice, Czechia; ^4^Faculty of Science, Department of Botany, University of South Bohemia, České Budějovice, Czechia

**Keywords:** alpine ecosystem, β-diversity, climate change, Himalaya, mountain summits, species richness, thermophilization

## Abstract

Under the contemporary climate change, the Himalaya is reported to be warming at a much higher rate than the global average. However, little is known about the alpine vegetation responses to recent climate change in the rapidly warming Himalaya. Here we studied vegetation dynamics on alpine summits in Kashmir Himalaya in relation to *in situ* measured microclimate. The summits, representing an elevation gradient from treeline to nival zone (3530–3740 m), were first surveyed in 2014 and then re-surveyed in 2018. The initial survey showed that the species richness, vegetation cover and soil temperature decreased with increasing elevation. Species richness and soil temperature differed significantly among slopes, with east and south slopes showing higher values than north and west slopes. The re-survey showed that species richness increased on the lower three summits but decreased on the highest summit (nival zone) and also revealed a substantial increase in the cover of dominant shrubs, graminoids, and forbs. The nestedness-resultant dissimilarity, rather than species turnover, contributed more to the magnitude of β-diversity among the summits. High temporal species turnover was found on south and east aspects, while high nestedness was recorded along north and west aspects. Thermophilization was more pronounced on the lower two summits and along the northern aspects. Our study provides crucial scientific data on climate change impacts on the alpine vegetation of Kashmir Himalaya. This information will fill global knowledge gaps from the developing world.

## Introduction

Biological consequences of climate warming are becoming increasingly obvious across a wide range of ecosystems (Woodward et al., 2010; Bellard et al., 2012; Grimm et al., 2013; García et al., 2018). Alpine ecosystems in particular are considered to be highly sensitive to global warming as they are generally adapted to lower temperature regimes (Körner, 2003; Chersich et al., 2015; Elmendorf et al., 2015). Despite their extreme environmental conditions, alpine ecosystems harbor rich biodiversity with a high degree of endemism (Gehrke and Linder, 2014; Smyčka et al., 2017; Noroozi et al., 2018). In recent decades, alpine ecosystems are reported to experience relatively higher rates of warming under anthropogenic climate change (Lenoir et al., 2008; Gottfried et al., 2012; Gobiet et al., 2014; Steinbauer et al., 2018). Recent warming trends recorded in alpine landscapes have resulted into species’ range shifts and changes in taxonomic and functional diversity, including the plant colonization of newly deglaciated areas at higher altitudes (Alexander et al., 2015; Steinbauer et al., 2018). The climate warming is expected to change alpine plant communities toward increasing dominance of warm-adapted species and loss of cold-adapted species, a phenomenon referred to as *thermophilization* (Gottfried et al., 2012; De Frenne et al., 2013). Thus, plant species which are narrowly specialized to cold habitats move upwards along elevation or experience local extinctions (Pauli et al., 2012; You et al., 2018). Therefore, alpine ecosystems can serve as best natural experimental systems to investigate the climate change-induced impacts on biological communities (Grabherr et al., 2010; Malanson et al., 2011), and these relatively pristine ecosystems can provide credible scientific evidence for detection of initial-warning signals of climate change (Wolf et al., 2012).

Across the globe, especially in Europe and North America, alpine summits have recently received research resurgence of studies addressing the warming-induced changes in biodiversity (Gottfried et al., 2012; Pauli et al., 2012; Grytnes et al., 2014; Vanneste et al., 2017; Steinbauer et al., 2018). Few studies have reported species’ range shifts on alpine summits (Chen et al., 2011; Grytnes et al., 2014). These changes in biodiversity on summits have highlighted that alpine flora is highly sensitive to rising temperatures (Bertrand et al., 2011; Gottfried et al., 2012; Vanneste et al., 2017; Steinbauer et al., 2018). In alpine areas, despite the availability of local biotic and abiotic refuges (Scherrer and Körner, 2011; Anthelme et al., 2014), the plant species need to move upward to adapt to the direct and indirect effects of warming (Lenoir et al., 2008; Gottfried et al., 2012; You et al., 2018). Alpine plant species are particularly sensitive to these range shifts because newly available upper habitats are devoid of life (Zimmer et al., 2018). Moreover, the migration lag during upward shifts experienced by alpine plants, i.e., the time between a climatic fluctuation and the point when plants successfully colonize a new site, has been shown to significantly affect plant distributions in mountainous areas (Dullinger et al., 2012). The contemporary increasing trend of temperature should therefore also affect the velocity of changes observed in alpine summit flora (Smith et al., 2015). Effects of accelerated global warming on alpine summit flora can only be recognized by long-term *in situ* monitoring. The mountain summits are ideal for long-term studies of plant responses to climate change because they represent prominent landmarks which can easily be located on subsequent surveys and future re-surveys (Pauli et al., 2015). In particular, gradients in biotic and abiotic conditions in mountainous areas are considered to have a substantial potential to explain species distributions and species richness patterns (McCain and Grytnes, 2010).

Although several factors have been reported to influence species richness patterns in alpine region (Vetaas et al., 2019), yet the most prominent driver of species’ re-distribution and range shift is climate warming (Gottfried et al., 2012; Pauli et al., 2012; Grytnes et al., 2014; Lamprecht et al., 2018). A recent meta-analysis by Steinbauer et al. (2018) on the alpine mountain summits across Europe has clearly demonstrated warming-induced changes in plant species richness. In fact, interactions of temperature and precipitation have been reported to influence responses of alpine plants to changing climatic conditions (Rapacciuolo et al., 2014; Vetaas et al., 2019). However, the changes in alpine plant species in response to recent warming in the moisture-limited Mediterranean mountains differ from shifts seen in the temperature-limited temperate mountains (Pauli et al., 2012). The patterns of plant species richness on mountain summits could also be affected by changes in grazing and tourism intensities (Walther et al., 2005). Local studies have suggested that frequent disturbance by tourists (Gottfried et al., 2012) and grazing (Speed et al., 2012) may suppress the elevational advance of alpine plants in response to climate warming in mountains. Land-use changes may thus explain part of the local variation in species richness trends, but they vary greatly within and among regions (Steinbauer et al., 2018). Therefore, it is of interest to understand the sensitivity of alpine vegetation to climate warming at regional scale and also in regions where risen temperature coincides with reduced precipitation (Engler et al., 2011).

While the accumulated scientific knowledge on alpine environments is growing fast, there are still substantial knowledge gaps across the world. Though some ambitious research efforts have been made in studying boreal and temperate alpine ecosystems, mainly in the northern hemisphere, some important alpine areas of the world, such as the Himalaya, have received little research attention. The Himalaya, sustaining the world’s highest mountain peaks, undoubtedly is one of the most sensitive areas to climate warming (Immerzeel et al., 2010; Rowan, 2017; Shekhar et al., 2017). The Himalaya, being one of the global biodiversity hotspots, harbors diverse alpine flora (Myers et al., 2000; Dar and Khuroo, 2013). Under the contemporary climate change, this region is believed to be warming at a much higher rate than the global average (Kumar et al., 2006).

Kashmir, a mountain region nestled in the north-western extreme of the Himalaya, has experienced a significant influence of global climate change over the last few decades (Immerzeel et al., 2010; Romshoo et al., 2015; Murtaza and Romshoo, 2017; Romshoo et al., 2017). Based on long-term records of temperature and precipitation, Zaz et al. (2019) recently reported a rise of 0.8°C in average annual temperature over the last half a century in Kashmir Himalaya. The study also reported a relatively greater warming (1.04°C) and decrease in annual precipitation (−16.7 mm year^–1^) at high-altitude areas, like Gulmarg (i.e., study area of present work), as compared to lower altitudes in this Himalayan region. However, it is unknown whether such climate change-induced warming has any impact (for example, changes in species composition, or range) on the biodiversity of this region. Moreover, most data on vegetation responses to warming come from long-term monitoring studies that began earlier in the 20th century (Holzinger et al., 2008) and encompass both relatively warmer and cooler periods through the last century. These effects of long-term climate changes may interact with other human-induced effects, e.g., effects of land-use change, or those of pollution. Hence data showing shifts in alpine vegetation may at least partially reflect changes in factors other than climate. To avoid possibly biased conclusions due to confounding factors, we focused on vegetation responses over a shorter time period and in pristine alpine areas to clearly identify temperature effects on vegetation changes. The present study was carried out at the recently established long-term alpine biodiversity monitoring site at Gulmarg, Kashmir Himalaya by adopting GLORIA Multi-Summit Approach (Pauli et al., 2015). Specifically, we tested the following hypotheses: (i) vascular plant species richness, vegetation cover and soil temperature decrease with elevation on the mountain summits in Kashmir Himalaya, (ii) thermal differences among the mountain summits determine the patterns and change in species richness, (iii) β-diversity (species turnover and nestedness) vary both spatially and temporally on the studied summits, and (iv) the summit vegetation has shifted to more dominance of warm-adapted species (thermophilization).

## Materials and Methods

### Study Area

**FIGURE 1:**
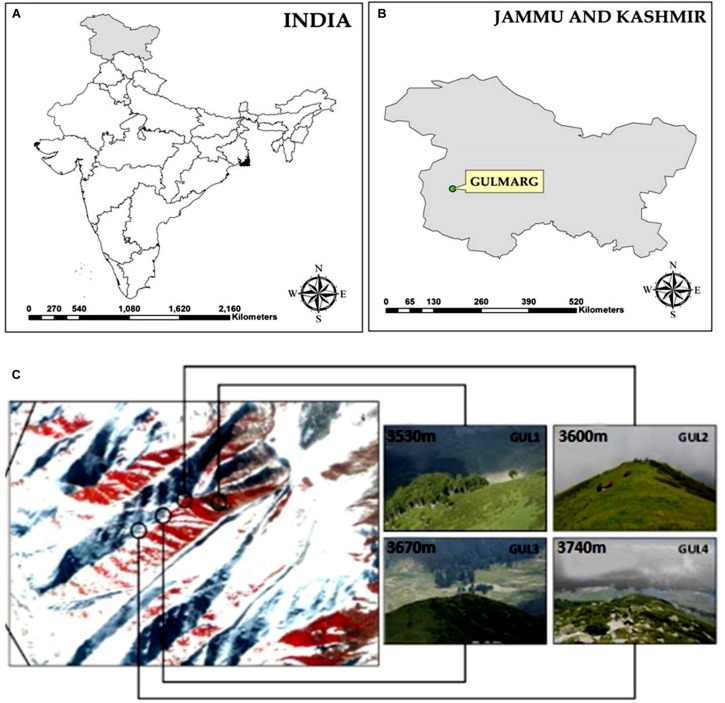
**(A,B)** Location map of study area, **(C)** a snapshot of the four studied summits.

The present study was conducted at Apharwat Mountain in Gulmarg, Jammu & Kashmir, which is located toward the North-Western Himalaya in India (Figures 1A,B). The study area is situated between 34°05′N latitude and 74°38′E longitude in the Pir Panjal range of the Himalaya. It encompasses the upper catchment area of Ferozpur stream and forests that surround the Gulmarg meadow.

Four summits were selected using the GLORIA protocol (Global Observation Research Initiative in Alpine Environments) representing an elevation gradient: GUL1 (3530 m), GUL2 (3600 m), GUL3 (3670 m), and GUL4 (3740 m) (Figure 1C). These summits are located between the lower alpine zone (close to treeline), to the higher alpine zone (close to nival zone). The climate of the study area is continental temperate, with annual average precipitation 1049 mm yr^–1^. The warmest month of the year is July with the temperature rising to an average of 20°C. January is the coldest month with the temperature going down to −6°C^1^. Dominant vegetation on the selected mountain summits comprise of plant assemblages belonging to *Rhododendron-Juniperus* and *Sibbaldia-Polygonum* patches. Besides, perennial grasses and sedges represent a good proportion on the summits.

### Vegetation Sampling

Standard GLORIA Multi-Summit Approach was followed to set up the monitoring sites at each summit (Pauli et al., 2015). Four 3-m^2^ permanent quadrat clusters along all the four mountain aspects (North, South, East, and West) were established at 5 m below the HSP (Highest Summit Point) (Supplementary Figure S1). Each 3-m^2^ quadrat clusters consisted of nine 1-m^2^ quadrats and whereof vegetation was recorded in the four corner 1-m^2^ quadrats only (filled quadrats in Supplementary Figure S1). This yielded vegetation data for 16 quadrats of 1-m^2^ per summit, and 64 quadrats in the four studied summits. For each 1-m^2^ quadrat, a complete list of vascular plant, lichen and bryophyte species was recorded and the cover of the surface types (rock, scree) was visually estimated. The percentage cover of each species was estimated using a percentage scale relative to the total quadrat area of 1-m^2^.

In addition to the establishment of 3-m^2^ permanent quadrats, each summit was divided into eight summit area sections: two (an upper and a lower section) along each aspect (Supplementary Figure S1). A string (contour line) around the summit, connecting the eight lower corner points of four 3-m^2^ quadrats at the 5 m level from the HSP delimits the upper summit area (= 5 m summit area) (Supplementary Figure S1). The 5 m summit area also includes the 3-m^2^ quadrats. Similarly, the corner points at the 10 m level from HSP mark the lower limit of the lower summit area (= 10 m summit area), which forms a zone around the 5 m summit area (Supplementary Figure S1). The 10 m summit area does not include (or overlap with) the 5 m summit area. All the vascular plant species were recorded in each summit area section and their percentage cover was estimated visually using five abundance classes (Supplementary Table S1).

Sixteen soil temperature data loggers (Make GEO-Precision M-Log5 W logger) were installed at 10 cm soil depth in order to record the soil temperature at 1 h interval. At each summit, four data loggers were installed; one in the middle of each of the 3-m^2^ quadrat clusters (Supplementary Figure S1).

First sampling of vegetation data was conducted in the 3rd week of August, 2014. A re-survey of the permanent plots was conducted after 4 years during the 3rd week of August in 2018.

### Climate Data

Precipitation and temperature data of Gulmarg for the period 2014–2018 were obtained from India Meteorological Department, Srinagar^2^. The mean monthly temperature was further analyzed for showing monthly fluctuation in weather parameters. Additionally, sixteen temperature data loggers installed at root zone (10 cm depth) provided crucial data to measure the micro-site level spatial and temporal variation of soil temperature on the studied summits.

### Data Analyses

All the statistical analyses were conducted in R version 3.5.2 (R Core Team, 2019). Temporal changes in air and soil temperature were analyzed with linear regression. Further, from each temperature logger, we first calculated daily average of hourly values as Tavg = 0.5 × (Tmin + Tmax), where Tmin and Tmax are the daily minimum and maximum temperatures, respectively. Growing Degree Days (GDD) were then calculated from August 2014–July 2015 and August 2017–July 2018 (as data loggers were installed in August 2014) using the following formula:

GDD=Tavg-5

Threshold value of 5°C was used since it has been reported to be most justified biologically for alpine ecosystems (Scherrer and Körner, 2011). GDD were calculated by considering all the days during which Tavg was above 5°C. Moreover, due to battery failure of two loggers out of 16, a 20 day data gap was created in each of these two loggers until we successfully replaced the battery. Since the loggers were installed on different cardinal directions of the summits with relatively different microclimates, to avoid possibly biased estimates and to get the true picture of the aspect-wise temperature variation of the studied summits, we preferred to fill these gaps based on the recorded temperature of the previous year in the same logger using R package *Amelia II* (Honaker et al., 2011). The package uses an EM (expectation maximization) algorithm on multiple bootstrapped samples of the incomplete original dataset. Imputation was repeated 30 times. Finally differences in GDD among the two years were assessed with ANOVA.

To determine how species richness (i.e., the number of species) respond to aspect and summit, a one-way ANOVA test was done with “aspect” (nested within summit) and “summit” as fixed effects, and the number of species per quadrat as response variables. Further, a multiple pairwise-comparison between the aspects and summits separately was performed with a Tukey multiple comparison test using *multicomp* package in R (Torsten et al., 2008) to determine whether the mean difference between the specific pairs of groups is statistically significant.

Next, to determine how species richness changed over time, a two-way ANOVA test was performed with “sampling year,” “summit” and their interaction as fixed effects, and the species number per quadrat as response variable. This procedure was repeated for soil temperature, specifying “aspect” and “summit” as fixed effects and soil temperature as response variables. Prior to ANOVA test, data was subjected to Shapiro-Wilk and Levene’s tests for checking the normality and homogeneity of variance, respectively. Levene’s test was performed using *leveneTest* function in R package *car* (Fox and Weisberg, 2019). Levene’s test is considered to be more robust in the checking the homogeneity of variance as it is less sensitive to departures from normal distribution.

### Changes in Species Richness, Cover Percentage

We calculated mean number of species, cover percentage of each species and mean soil temperature. For species richness and cover both large-scale (summit area sections) and small-scale (species per 1-m^2^ quadrat) data were used in these calculations. The temporal change for species richness and cover percentage between 2014 and 2018 was then calculated by simple subtraction. The statistical significance of these differences were then analyzed by ANOVA. Next, boxplots, and barplots were prepared using R package *ggplot2* (Wickham, 2016).

Further, to determine the effect of variables such as elevation, aspect and year of sampling on species richness and cover percentage, variation partitioning was used (Heikkinen et al., 2005). Variation partitioning was performed using the *varpart* function in R package *vegan* (Oksanen et al., 2019). The method splits the total variation into seven fractions: (i) pure effect of elevation (E), (ii) pure effect of aspect (A), (iii) pure effect of year of sampling (Y), and shared variation due to each pair (iv) elevation and aspect (E∩A), (v) elevation and year of sampling (E∩Y), (vi) aspect and year of sampling (A∩Y), plus combined effect of all three variables (vii) elevation, aspect and year of sampling (E∩A∩Y). Statistical significance was then evaluated using Monte Carlo permutation test (number of permutations = 999). Since shared effects were obtained by subtraction, they could not be tested for significance (Legendre and Legendre, 2012).

### β-Diversity

We calculated the turnover (species replacement between summits) and nestedness (species gain or loss between summits) components of beta diversity to examine spatial patterning of turnover and nestedness-resultant dissimilarity among summits using a *betapart* package (Baselga and Orme, 2012).

This package partitions the pairwise Sorenson dissimilarity between the two sites (βsor) (Equation 1) into two additive components (Equation 2) accounting for species spatial turnover (βsim) (Equation 3) and nestedness-resultant dissimilarities (βsne) (Equation 4) (Baselga, 2012).

(1) βsor=b+c/2a+b+c

(2) βsor=βsim+βsne

(3) βsim=min(b,c)/a+min(b,c)

(4) $$\begin{array}{c} \beta sne=\beta sor-\beta sim\\ =b-c/2a+b+c\times a/a+min\left(b,c\right) \end{array}$$

where, “a” is the number of species present at both sites, “b” the number of species at first site but not at second, “c” is the number of species present at second site but not at first. Finally, cluster analysis was done based on dissimilarity matrices yielded between the sites (here aspects of summits) using R package *vegan* (Oksanen et al., 2019).

We also assessed temporal differences in species composition among four different aspects (nested within summit) and relative contribution of nestedness and turnover. Using the command *beta.tem* from the R package *betapart*, variation in species composition across time was measured as the dissimilarity between 2014 and 2018.

### Thermophilization

We quantified thermophilization to assess the effect of climate warming in species composition on the studied summits. Here, a change in elevation (from GUL1-GUL4) was considered to consistently represent a thermal gradient. Every documented vascular plant species received an elevational rank (Supplementary Tables S2, S3), which was mainly based on species’ location on each summit, augmented with local floras (Dhar and Kachroo, 1983; Polunin and Stainton, 1984). The thermic vegetation indicator (*S*) was then calculated using the following formula:

$$\begin{array}{c} S=\left(\Sigma rank\left(species_{i}\right)\times cover\left(species_{i}\right)\right)/\\ \;\Sigma cover\left(species_{i}\right)\;\left(\mathrm{Gottfried et al.,}\;2012\right) \end{array}$$

Subsequently, thermophilization indicator (D) was calculated for 2014 and 2018 as shift over time (D = *S*_2018_−*S*_2014_, positive difference denote an increasing thermophilization). Finally, to determine whether thermophilization indicators differed significantly from zero, Wilcoxon singed rank test was performed.

We also compared plant distribution data collected in 2014 with recent re-surveyed (2018) data recorded in the same permanent plots, to evaluate changes in species composition.

## Results

### Temporal Changes in Climatic Variables

Overall, the period studied was characterized by reduced precipitation and warming temperature. Climate records from our study area during 2014 to 2018 showed an increase of 0.4°C in average annual temperature, with higher increase in maximum temperatures compared to minimum temperatures. A significant increase in monthly mean temperature for January, February, March, April, July, and August from 2014 to 2018 was observed (Linear regression, *F* = 9.89, *df* = 1, *P* = 0.005) (Supplementary Figure S2A). However, a decreasing trend for precipitation was observed (Linear regression, *F* = 7.82, *df* = 1, *P* = 0.003). The decrease in mean annual precipitation from 2014 to 2018 amounted to −38.9 mm, and a significant decrease in precipitation was observed for winter (January, February, and March) (Linear regression, *F* = 5.23, *df* = 1, *P* = 0.005) and summer months (July and August) (Linear regression, *F* = 8.05, *df* = 1, *P* = 0.003) (Supplementary Figure S2B). The annual mean soil temperature increased by 1.5°C from 2014 to 2018 at our summits, with more increase in winter (December, February, March), spring (April, May) and autumn months (September, October) (Linear regression, *F* = 8.76, *df* = 1, *P* = 0.006) (Supplementary Figure S3). In addition, growing degree days increased on all of the studied summits from the period August 2014–July 2015 to the period August 2017–July 2018, but the increase was statistically insignificant (ANOVA test, *P* = 0.823, Supplementary Tables S4, S5).

### Patterns of Species Richness, Cover Percentage and Soil Temperature

**FIGURE 2:**
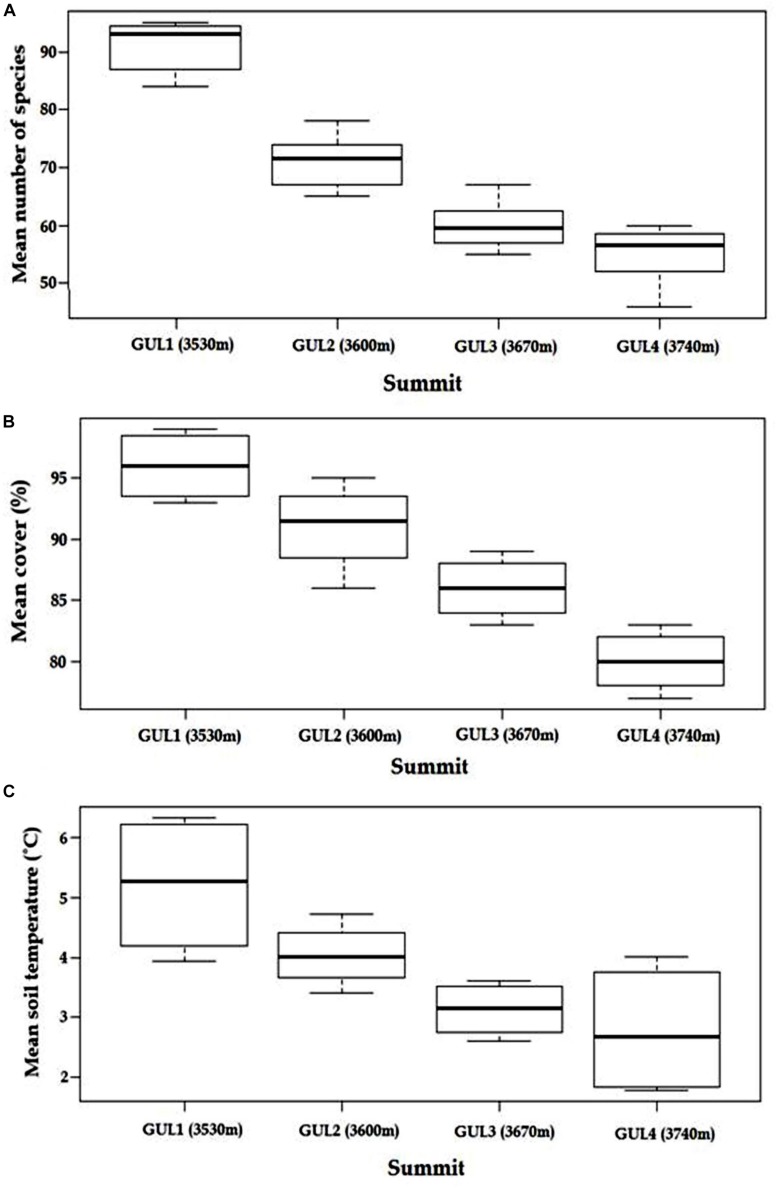
Patterns of **(A)** mean species richness, **(B)** mean cover percentage and **(C)** mean soil temperature with successively increasing elevation of summits in 2014. Results are depicted as mean ± standard error of mean. Whiskers in the box plot indicate the 25 and 75% percentiles. The line within each boxplot represents the mean value for that plot.

The initial survey in 2014 revealed that, with an increase in elevation, species richness, cover percentage and soil temperature decrease (Figures 2A–C). Between the lower elevation GUL1 and GUL2 summits (first lag), there is an elevational increase of 70 m, 22% decrease in the species richness, and a 1.2°C decrease in the soil temperature, whereas between GUL2 and GUL3 (second lag) the elevational increase is again 70 m but decrease in species richness and temperature was only 15.5%, and 0.9°C, respectively. Similarly, between the higher elevation GUL3 and GUL4 summits (third lag), again the elevation increase is 70 m but only 8.3% decrease in species richness and 0.3°C decrease in temperature was found. Thus both species richness and temperature dropped more rapidly in the elevation zone from 3530 m (GUL1) to 3600 m (GUL2). Overall, from the lowest summit (GUL1, 3530 m) to the highest summit (GUL4, 3740 m) there is an elevational increase of 210 m and decrease in species richness and soil temperature by 39.6% and 2.4°C, respectively. Similar trend was observed for cover percentage. The lowest summit GUL1 showed vegetation cover of 96%, followed by GUL2 with vegetation cover of 91%. GUL3 summit showed vegetation cover of 86%, and the highest summit GUL4 showed vegetation cover of 80%.

### Aspect-Wise Species Richness and Soil Temperature

**FIGURE 3:**
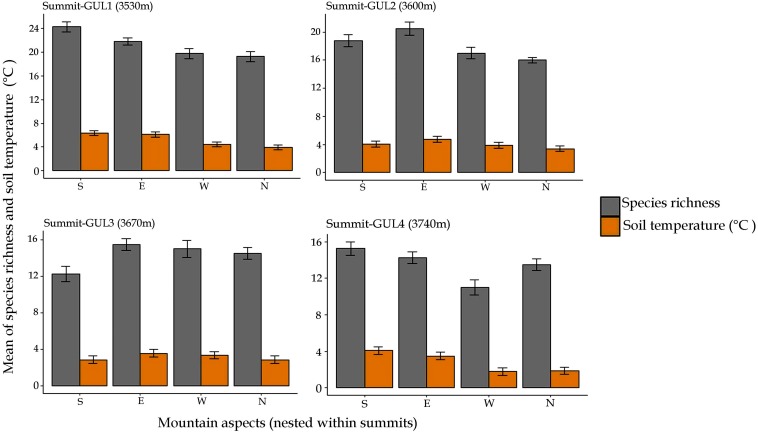
Patterns of mean of species richness and soil temperature with aspect in 2014. Results are depicted as mean ± standard error of mean.

The patterns of species richness and soil temperature for the year 2014 were also determined by aspect as well (Figure 3). Our results revealed that there is direct relation between species richness and soil temperature. We observed that the aspect with higher soil temperature favored more local-scale species richness compared to the aspect with lower soil temperature of the same mountain summit (Figure 3). For instance, at GUL1, the south aspect had the highest average annual soil temperature (6.3°C) and was found to have also the highest species richness; whereas at GUL2, the north aspect had the lowest soil temperature (3.4°C), and was found to have the lowest value of species richness. Similarly, at GUL3 and GUL4 east and south aspects respectively were found to be warmer (3.6, 4.1°C), and have correspondingly higher species richness. Whereas, south and west aspects at GUL3 and GUL4 summits were coldest (2.9 and 1.8°C, respectively), and had accordingly lower values of species richness (Figure 3).

### Temporal Changes in Species Richness and Cover Percentage

**FIGURE 4:**
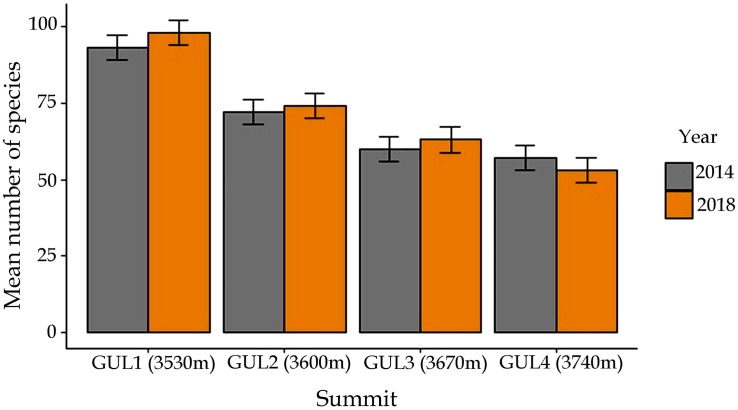
Mean number of species in the two study years 2014 and 2018. Results are depicted as mean ± standard error of mean.

A comparison of species richness between 2014 and 2018 revealed that the number of vascular plant species on all the summit areas combined increased from 138 to 146. In 2014, the total number of plant species recorded was 92 on GUL1, 68 on GUL2, 58 on GUL3 and 55 on GUL4 (Figure 4). The net increase in the number of plant species from 2014 to 2018 was 5.4% on GUL1, 4.4% on GUL2, and 3.4% on GUL3. However, on the highest summit there was a 5.4% decrease in the species number from 2014 to 2018.

While the total number of plant species increased on summits from 2014 to 2018, no significant change was found at a smaller spatial scale, i.e., at the 1-m^2^ quadrat level. However, at this spatial scale we observed a significant increase of total cover of shrubs, forbs and graminoids from 2014 and 2018 on all of the four summits (Table 1). The percentage cover of the most dominant shrub species (*Cotoneaster microphyllus, Gaultheria trichophylla, Juniperus squamata, Lonicera obovata*, and *Rhododendron anthopogon*), graminoid species (*Poa angustifolia, Polypogon fugax*, and *Themeda anathera*) and forbs (*Polygonum affine*, *Sibbaldia cuneata*, and *Swertia petiolata*) on studied summits is shown in the Table 1.

**TABLE 1:** Temporal changes in cover percentage of most dominant shrubs, forbs and graminoids from 2014 to 2018 ± standard error of mean (SEM) in 1-m^2^ quadrats on the four studied summits.

Species	Change in cover (%)	*P*-value
**Shrubs**		
*Cotoneaster microphyllus* Wall. ex Lindl.	2.80 ± 0.876	0.003
*Gaultheria trichophylla* Royle	1.50 ± 0.133	<0.005
*Juniperus squamata* Buch.-Ham. ex D.Don	3.05 ± 0.987	0.007
*Lonicera obovata* Royle ex Hook.f. & Thomson	0.92 ± 0.192	0.002
*Rhododendron anthopogon* D. Don	0.80 ± 0.199	<0.001
**Graminoids**		
*Poa angustifolia* L.	1.20 ± 0.198	0.003
*Polypogon fugax* Nees ex Steud.	1.30 ± 0.268	0.004
*Themeda anathera* (Nees ex Steud.) Hack.	0.5 ± 0.282	0.001
**Forbs**		
*Polygonum affine* D. Don	2.02 ± 0.534	<0.003
*Sibbaldia cuneata* Edgew.	3.09 ± 0.999	0.002
*Swertia petiolata* D. Don	1.50 ± 0.133	<0.001

### Effect of Summit, Aspect and Year on Species Richness and Soil Temperature

The results of one-way ANOVA test revealed a significant effect of aspect and summit on species richness (Supplementary Tables S6–S10). Moreover, a two-way ANOVA test revealed a significant interaction of summit and year on species richness (*P* < 0.0404) (Supplementary Table S11). Similarly, a significant effect of summit and aspect on soil temperature was also observed (Supplementary Table S12).

In variation partitioning of species richness, the total variation explained by elevation, aspect and year of sampling was 68.5% (Table 2). Of the pure effects, elevation (E) was found to be the most important variable for determining species richness on the summits. The pure effects of aspect (A) and year of sampling (Y) variables were small, but statistically significant. Among the shared effects, (E∩A∩Y) and (E∩A) were the most important components. Similarly, for cover percentage the total variation explained was 48.7% (Table 2). A pure effect of elevation (E) was again the most important component, but pure effect of year of sampling (Y) gained importance in explaining the cover percentage. Further, shared effects of (E∩A∩Y) and (E∩Y) were important components in determining cover percentage on summits.

**TABLE 2:** Variation partitioning of plant species richness and cover percentage into effects of elevation (E), aspect (A), and year of sampling (Y) variables (adjusted *R*^2^ in%).

	Pure effects			Shared effects				Total variation explained
	E	A	Y	E∩A	E∩Y	A∩Y	E∩A∩Y	
Species richness	10.2 (0.03)	5.4 (0.01)	3.2 (0.04)	15.2	11.2	5.8	17.5	68.5 (0.01)
Cover percentage	7.2 (0.01)	2.3 (0.01)	5.5 (0.02)	6.5	10.3	2.6	14.3	48.7 (0.01)

*The p-values of pure effects are shown in brackets {p < 0.05; Monte Carlo permutation test (number of permutations = 999)}.*

### Spatio-Temporal Patterns in β-Diversity

**FIGURE 5:**
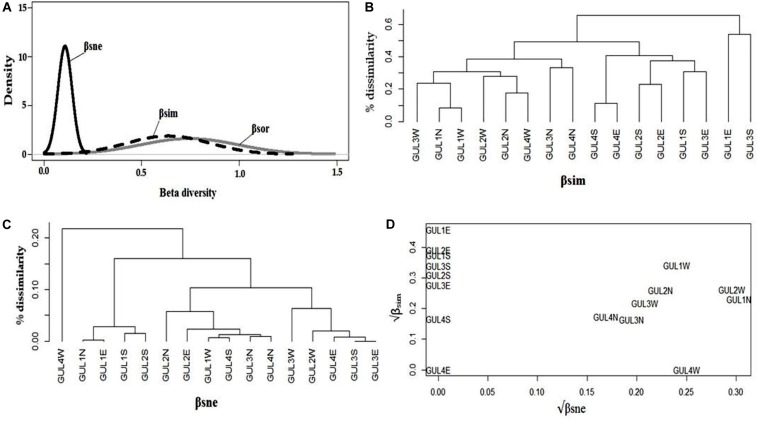
Multiple-site dissimilarities across the four studied summits GUL1 – GUL4 and the four aspects north (N), south (S), east (E), and west (W) on each of the summits. **(A)** Partitioning of βsor (total dissimilarity – gray line) into βsim (turnover or species replacement component of beta diversity – dashed line) and βsne (nestedness or richness difference component of beta diversity – solid line) for the study summits and aspects. Average clustering of **(B)** βsim and **(C)** βsne components of species dissimilarity among the summits and aspects. **(D)** Comparison of the square root transformed βsim and βsne components of βsor between 2014 and 2018 for the summits and aspects. In **(B**–**D)**, the acronyms indicate the combination of summit and aspect.

We analyzed the multiple-site dissimilarities and found that multiple-site Sørensen dissimilarity among all the studied summits was comparatively low. The nestedness component (β_sne_) was found to be the largest contributor to the overall dissimilarity. Note that despite more or less similar values of total dissimilarity (β_sor_) and turnover (β_sim_), nestedness-resultant dissimilarity (β_sne_) is higher (Figure 5A). It indicates that dissimilarity among summits is mostly because of richness difference (nestedness) and less importantly because of species replacement (turnover).

We analyzed pairwise dissimilarities among aspects of the four summits to disentangle the patterns of dissimilarity derived from turnover, and those derived from nestnedness. Cluster analysis derived from the dissimilarity matrices of turnover component revealed that east aspect of GUL1 and south aspect of GUL3 are highly dissimilar from rest of the aspects, followed by north aspects of GUL3 and GUL4 (Figure 5B). Contrary to the results obtained with species turnover, cluster analysis obtained from dissimilarity matrices of nestedness revealed a quite different pattern. Four distinct clusters were obtained. The west aspects of GUL3 and GUL2, east aspects of GUL3 and GUL4, and south aspect of GUL3 are quite dissimilar from the rest of summit aspects. It is important to notice that based on the nestedness-resultant dissimilarity, the west aspect of GUL4, which falls in the sub-nival zone, was highly dissimilar from the other aspects of the four summits (Figure 5C).

The temporal variation between 2014 and 2018 of β-diversity among different aspects of the four summits revealed a significant contribution of both of the components of β-diversity to the overall dissimilarity (Figure 5D). The results revealed that the south and east aspects of all of the summits showed only replacement of species (species turnover) between 2014 and 2018, with highest turnover observed for east aspect of GUL1, thereby implying that no significant species loss or gain has occurred on these aspects during a span of five years. In contrast, the north and west aspects of all of the summits showed both species turnover and nestedness, with highest values observed for north and west aspects of GUL1 and GUL2 (Figure 5D). Moreover, the overall dissimilarity among these aspects was dominated by nestedness-resultant dissimilarity. Importantly, the west aspect of GUL4 showed high nestedness-resultant dissimilarity (β_sne_) and zero turnover (β_sim_), thereby implying that at this aspect relatively higher loss or gain of species occurred without any replacement of species between 2014 and 2018; while the east aspect of GUL4 remained at a steady state during the four years (Figure 5D).

### Thermophilization

**FIGURE 6:**
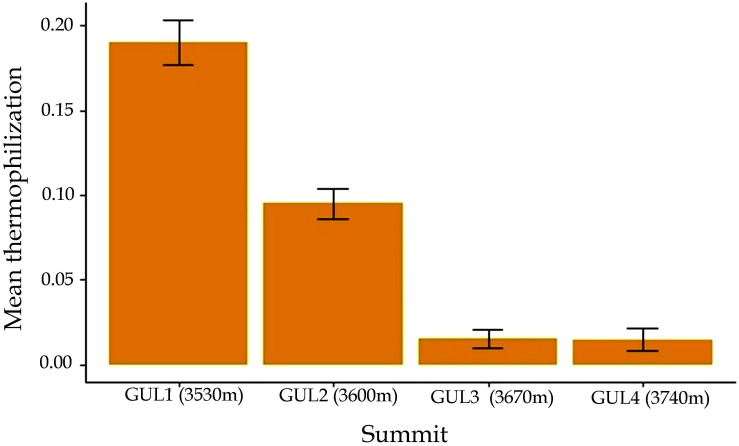
Thermophilization indicator from 2014 to 2018 in all of the 1-m^2^ quadrats at each of the four studied summits. Results are depicted as mean ± standard error of mean.

Our results indicated thermophilization at the lower two summits between 2014 and 2018 (Wilcoxon signed rank test, *P* = 0.004), whereas at the upper two summits, thermophilization was negligibly small (Figure 6). A comparison of plant distribution data recorded in 2014 and 2018 revealed that some plant species present in the lowest summit GUL1 in 2018, such as *Aquilegia fragrans*, *Cimicifuga kashmiriana, Cirsium wallichii*, *Impatiens sulcata*, and *Picris hieracioides* were not reported on this summit in 2014. Similarly, on the GUL2 summit, species like *Delphinium vestitum*, *Erigeron multicaulis* and *Nepeta linearis* were found for the first time in 2018. On GUL3, plant species such as *Impatiens brachycentra* and *Mazus pumilus* were found to have newly appeared in 2018. However, the three species *Corydalis cashmeriana, Elsholtzia eriostachya*, and *Silene himalayensis*, which were reported on the highest summit GUL4 in 2014, were absent in 2018.

## Discussion

Contemporary climate warming is considered as the key driver of recent shifts in alpine plant distributions (Gottfried et al., 2012; De Frenne et al., 2013; Steinbauer et al., 2018). The present study provides empirical evidence to substantiate the sensitivity of alpine vegetation in the Himalaya to the ongoing climate change. The increase in species richness on our studied summits reveal that climate-induced biotic change is occurring even at the world’s upper limit of vascular plant life, with possibly significant consequences not only for biodiversity, but also for ecosystem functioning and services.

### Patterns of Climate Change

Based on the *in situ* data recorded during the present study, increase in mean annual temperature, and a decrease in precipitation was clearly observed. An almost similar trend was reported in the analysis of historical climate data by Zaz et al. (2019) in this Himalayan region. In Himalayan mountains, different trends have been observed in precipitation over recent decades (IPCC, 2001). While some studies (Shrestha, 2000; Borgaonkar and Pant, 2001) observed increasing precipitation, others reported a decreasing precipitation trend (Kumar and Jain, 2010; Dimri and Dash, 2012; Zaz et al., 2019). The present study found higher warming and decreasing precipitation trends in winter and spring months, which is consistent with other studies carried out both in the same region (Zaz et al., 2019) and in other parts of Himalaya (Archer and Fowler, 2004). The mean annual temperature and the monthly mean temperatures increased in our summits. This is consistent with other air temperature records during the same period. Since external factors, such as air temperature, relative humidity, percent of cloud cover, precipitation, solar radiation etc. all contribute to variation in soil temperature (Walsh et al., 2007), it is likely that warming of air temperature combined with decrease in precipitation could have significantly increased soil temperature in our summits.

### Variation of Species Richness and Cover Percentage With Elevation

The present study demonstrated that the plant species richness and cover percentage decreased significantly toward higher elevations, which is in accordance with other studies (Grytnes et al., 2006; Kazakis et al., 2007; McCain and Grytnes, 2010; Erschbamer et al., 2011; Vanneste et al., 2017). The observed decrease of species richness along increased elevation supports the hypothesis put forward by Körner (2011). Reduction of the potential growth area and increase of the terrain slope with increased elevation would be plausible explanations of the concomitant reduced species richness with increased elevation (Grytnes, 2003; Theurillat et al., 2011). Moreover, a steeper terrain is more susceptible to erosion. Yet, another possible explanation could be the difference in soil temperature (for instance, demonstrated by the present study, through difference in soil temperature equal to 2.4°C between the lowest and the highest summit). Certainly, alpine plant life is influenced by temperature both directly through setting limits to species’ fundamental niches (Waldock et al., 2018), as well as indirectly through determining decomposition and nutrient cycling, access to water, and abundance of pathogens, pollinators, herbivores, and seed dispersers. Moreover, the dropping of temperature with increasing elevation also exerts a substantial influence on the pool of potential species that are able to thrive in the increasingly harsh conditions. Hence, composition of plant communities at higher elevations consists of a smaller sample of the regional species pool (Bruun et al., 2006).

Although the present study observed significant decline of both species richness and temperature with elevation, this decline showed the highest rates in the elevation zone between 3530 m (GUL1-close to treeline) and 3600 m (GUL2-alpine), which represents a direct transition from treeline to the alpine zone. This finding is in line with the study carried out by Kazakis et al. (2007) on White Mountains of Crete, Greece. Such a trend can be ascribed to the loss of vigorously growing low elevation montane-treeline species and gain of more slow growing alpine species along elevation, in combination with relatively low number of species in the increasingly harsh conditions at higher elevations of the alpine zone. Moreover, as GUL1 actually represents an ecotone between the treeline below and alpine meadows above it, some sort of edge effect of species assemblages between treeline and main alpine zone becomes obvious. Therefore, in view of projected trends of climate change, these middle zones (ecotones) are of immediate importance for identifying possible future boundary shifts of plant assemblages and for predicting the fate of species in the higher elevations.

### The Warmer, the Richer

Our study reveals a direct relation between species richness and aspect. The south and east mountain aspects favor local-scale species richness, compared to the north and west aspects at the same elevation, which largely confirms the results of other studies (Gutierrez-Giron and Gavilan, 2010; Winkler et al., 2016). However, this result contradicts the findings of Kazakis et al. (2007) who observed no direct relation between species richness and aspect. The effect of aspect can be attributed to the difference in soil temperature (as observed in the present study) among cardinal directions on mountain summits, with south and east aspects displaying higher temperatures than north and west aspects (Figure 3).

The most widely acknowledged abiotic environmental driver of plant life in alpine ecosystems is soil temperature (Körner and Hiltbrunner, 2018). Aspect causes pronounced differences in the thermal input, and therefore significantly influences species richness on mountain aspects. Several studies (Hawkins et al., 2007; Whittaker et al., 2007; Gillman et al., 2015; Winkler et al., 2016) have confirmed temperature and solar radiation as underlying determinants of species richness. In general, south and east aspects receive higher daily inputs of solar radiation compared to north and west aspects. As a result, temperature, soil moisture and nutrient cycling processes are affected. This has a substantial effect on local vegetation (Sternberg and Shoshany, 2001). Moreover, especially in temperate mountains convective cloud formation is a common phenomenon, which occurs most frequently after midday (Geiger, 1950). It largely decreases the daily direct radiation on the north and west aspects compared to south and east ones.

### Temporal Changes in the Summit Flora Between 2014 and 2018

The present study observed an increase in the species richness on summits during 4 years, and the increase was more pronounced on the lower summits. Our findings thus agree with the studies conducted in other parts of the world (Holzinger et al., 2008; Lenoir and Svenning, 2015; Dolezal et al., 2016; Lamprecht et al., 2018; Steinbauer et al., 2018). For instance, Steinbauer et al. (2018) also found an increase of species richness on 302 mountain summits across Europe over the past 145 years. Similarly, increase in vascular plant species richness of about 11% per decade in alpine zone of Alps was reported by Holzinger et al. (2008). Such an increase in species richness can be attributed to the warming-induced upward shift of species from lower elevations. Trait analyses from other studies provide clear evidence that new colonizers exhibit growth strategies characteristic of species from lower elevations (Dolezal et al., 2016). The present study thus suggests that increase in species richness on our selected mountain summits is a direct and instantaneous response to climate change and, thus, can be expected to be accelerated further, if climate warming continues at the same rate.

In the present study, a consistent increasing trend of vascular plant species richness was recorded in the lower three summits. However, the highest summit (which falls in the nival zone) showed an opposite trend, as a decline of species richness was observed on this summit between 2014 and 2018. It is likely that increased competition with lower elevation plants has excluded the species originally adapted to this summit and that local extinction was higher than colonization (Rumpf et al., 2019). This is because newly arrived plant species from lower elevations are potentially stronger competitors than cold-adapted nival species and possibly outcompete these species in resource utilization (Matteodo et al., 2013; Alexander et al., 2015). Therefore, as more species become established at high-elevation sites, local extinctions will likely be caused by competitive replacement of these slow-growing, stress-tolerant nival species by more vigorous low elevation generalists that benefit from warming (Alexander et al., 2015). However, at the same time, facilitation might offset the negative effects of competition (Callaway et al., 2002; Alexander et al., 2015). Facilitation, however, may not be immediately accompanied by a rapid species decline due to warming-induced habitat loss, because it could lag behind for several years, due to long-lived nature of most alpine plants (Dullinger et al., 2012). On the other hand, topographically diverse environments in alpine areas may further shield against the loss of climatically suitable habitats (Scherrer and Körner, 2011), but the warming may also reduce the availability of these fine-scale suitable habitats (Scherrer and Körner, 2011), as well as facilitate uphill movement of potential competitors, thereby increasing competitive pressure (Kulonen et al., 2018). These processes, along with species’ intrinsic ability to tolerate changing climates, may buffer against local extinctions, and a rapid loss of alpine-nival species may occur only later due to a rapid shrinking of climatically suitable area of alpine habitats under accelerated climate warming (Lamprecht et al., 2018). This loss of alpine-nival species will hardly be compensated soon because of the extremely slow growth at higher elevations (Dvorský et al., 2016). Consequently, the increase in species richness may be considered as a transient phenomenon that hides a more critical extinction rate.

The present study observed a significant increase in the coverage of most dominant shrubs, graminoids and forbs between 2014 and 2018. Our findings support several earlier studies (Grytnes et al., 2014; Jorgenson et al., 2015; Vanneste et al., 2017; Lemay et al., 2018). The increase in cover percentage can be attributed to the fact that the prevailing warming and drought conditions in the study area could have increased the length of growing season, thereby supporting colonization and vegetative spread. This hypothesis is supported by several authors (e.g., Gottfried et al., 2012; Pauli et al., 2012; Steinbauer et al., 2018), who indicated that climate induced responses of alpine plants are possibly due to combined effects of warming and a reduction in precipitation. In the present study, increase of the monthly mean temperatures and increased occurrence of dry conditions during spring and winter months could have caused faster melting of snow, thereby increasing the length of growing season in the study area. In alpine ecosystems, patterns of snow melt and growing season length have been reported to play important roles in determining patterns of species richness and vegetation spread (Wipf et al., 2009). The prolonged growing season can also pose considerable threat to high elevation plant species through the opening of immigration pathways for potentially stronger competitors from lower elevations (Matteodo et al., 2013). Increased drought risk of alpine plant species caused by warming has already been reported in Europe over the past century (Lamprecht et al., 2018; Rumpf et al., 2019). As revealed by the present study, a similar development seems to be ongoing in the Himalaya as well.

### Patterns of β-Diversity

Our results showed that nestedness contributed more to beta diversity than turnover. This is due to the fact that nestedness is positively associated to differences in the available area along elevation gradients in mountain landscapes. The suitable area for plant growth decreases with increasing elevation (Theurillat et al., 1998), and this has considerable influence on species richness, which decreases with elevation. Therefore, we observed dissimilarity among summits was contributed more by richness difference (nestedness) and less by replacement of species (turnover). This also reveals that the summits in our study system are less heterogeneous and a relatively small pool of species is exclusive to each summit. The higher multiple-site nestedness-related dissimilarity among summits suggests a higher probability of local species extinction (Si et al., 2015).

The high turnover on the south and east aspects can be attributed to the fact that colonization in alpine ecosystems occurs more frequently at warmer south and east aspects than at north and west aspects (Wipf et al., 2013). Similarly, species turnover on the majority of historical summit observation sites in the Alps has been attributed to colonization rather than to local extinction of species (Wipf et al., 2013). Contrary to this, high nestedness-resultant dissimilarity observed in the present study among north and west slopes could have arisen due to disappearance of local species. Since north and west aspects usually contain more cold-adapted species due to lower temperatures, local extinctions are expected when these cold-adapted species are gradually outcompeted by warm-adapted species, whereas colonization does not occur more frequently at north and west aspects on the mountain summits (Winkler et al., 2016).

### Upward Shift of Thermophiles

In the present study, changes in vascular plant species composition on studied summits indicate a shift toward more thermophilic vegetation, and this phenomenon was more pronounced on the lower two summits. Such a thermophilization trend has been reported from studies in other parts of the world (Gottfried et al., 2012; Dolezal et al., 2016; Vanneste et al., 2017; Lamprecht et al., 2018). This observation is also in line with the hypothesis that most species will shift upwards as a consequence of climatic warming (Engler et al., 2011). Indeed, the climate has become warmer and precipitation has been reduced between the two surveys on the mountain summits studied in the present study. This finding is in line with Sigdel et al. (2018) who observed that upward shift rates of treeline is primarily mediated by interactions of precipitation and temperature.

Thermophilization was also recorded more pronounced for the north and west aspects of the summits. North and west aspects generally contain more cold-adapted species; therefore, thermophilization effects may be expected to be enhanced when these cold-adapted species will be competitively displaced by warm-demanding species from the lower elevations. This can likely be related to larger species cover changes and/or migration of species with a higher rank and a lower elevational range (warm-adapted species) and/or to the decline of species with a lower rank and a higher elevational range (cold-adapted species). In fact, range contractions of cold-adapted species have been recently reported from the Alps (Rumpf et al., 2018). Moreover, some studies have shown that different functional traits and species interactions slow down warming induced upward shifts of plant species (Alexander et al., 2015: Liang et al., 2016). Therefore, future studies combining species’ functional traits and species interactions may prove particularly useful for determining species range shifts under climate warming in the Himalayan region.

### Resilience to Climate Change

A considerable difference in the distribution of individual species along the elevation gradient confirmed the high resilience of some of the recorded species. For instance, certain forbs like *Aquilegia fragrans*, *Cimicifuga kashmiriana, Cirsium wallichii*, *Impatiens sulcata*, *Picris hieracioides, Trifolium pratense, T. repens, Verbascum thapsus* and shrub species like *Rosa webbiana, Salix denticulata* were restricted to the lowest summit GUL1. These species are generally warm-adapted and have an optimal performance in the treeline ecotone or in the lower alpine zone. Other plant species like *Berberis jaeschkeana, Cortia depressa, Cortusa brotheri, Cotoneaster microphyllus, Crucihimalaya himalaica, Delphinium vestitum*, *Erigeron multicaulis*, *Impatiens brachycentra, Jurinea dolomiaea, Lonicera obovata, Nepeta linearis*, and *Thalictrum alpinum* occur between the lower and higher alpine zone. Species like *Corydalis cashmeriana, Cremanthodium decaisnei, Elsholtzia eriostachya, Ligularia amplexicaulis*, *Ligularia fischeri*, and *Saxifraga hirculus* were restricted to the highest summit or sub-nival zone. These are typical high-altitude species, which are adapted to the extreme environmental conditions, such as low temperature and high radiation intensities. In addition, some of these species are marked by a rather narrow altitudinal range (Dhar and Kachroo, 1983; Polunin and Stainton, 1984) and are therefore more prone to local extinction as a result of changing environmental conditions. Since continuous warming could potentially lead to more thermophilization, thereby compelling the sub-nival specialists to decline at the alpine-nival ecotone. Other species, e.g., *Juniperus squamata*, *Rhododendron anthopogon, Polygonum affine*, *Sibbaldia cuneata*, and *Swertia petiolata* have broad altitudinal ranges (Dhar and Kachroo, 1983; Polunin and Stainton, 1984). In our study, these species were also found on each of the four summits. These species are commonly widespread or ubiquitous, which means they are adapted to a wide range of environmental conditions. We therefore expect these generalist species to possess relatively high resilience to climate change.

### Future Implications

Understanding biodiversity-climate change relationship is critical to forecast future biodiversity and vegetation feedbacks to climate. The present study empirically explored the vegetation dynamics on alpine mountain summits in Kashmir Himalaya in order to fill the knowledge gaps that stem from the limited research data on warming-induced biodiversity changes in rapidly warming Himalaya. We observed increase in species richness during the re-sampling of the alpine summits, thereby providing early evidence of shifts in alpine summit vegetation and supporting the hypothesis that interactions of temperature rise and reduced precipitation can induce upward migration of alpine species. Although an increase in species richness might sound positive as species enrichment, it is an equally alarming signal because as new thermophilic species become established at higher summits, local species extinctions will likely result from competitive displacement of cold climate specialists by potentially more vigorous lower elevation generalists that benefit from warming, rather than from habitat loss directly through warming. Therefore, increase in species richness is expected to be a transient phenomenon that hides the accumulation of extinction debt. The current biodiversity change in the alpine summits in Kashmir Himalayan mountain ecosystems can have rapid and widespread consequences for ecosystem functioning, which merits detailed investigation in near future. The novel research insights will provide crucial baseline data to undertake qualitative/quantitative analyses of vegetation-climate dynamics in the Himalaya. More importantly, re-sampling of the summits in near future will furnish robust results on the impacts of climate change on alpine plant diversity in this ecologically fragile Himalayan region.

## Data Availability Statement

All datasets generated for this study are included in the article/Supplementary Material.

## Author Contributions

CS and AK developed the research idea. MH, AK, AM, RA, CS and SH collected field data. MH, RA performed statistical analyses with inputs from JD. MH led the manuscript writing with inputs from AK, JD. All of the authors approved final draft of the manuscript submission.

## Disclaimer

Frontiers Media SA remains neutral with regard to jurisdictional claims in published maps and institutional affiliations.

## Conflict of Interest

The authors declare that the research was conducted in the absence of any commercial or financial relationships that could be construed as a potential conflict of interest.

## Footnotes

1 http://www.imd.gov.in/pages/main.php

2 http://www.imd.gov.in/pages/regional_weather.php
