# Supplementary material for: Early Evidence of Shifts in Alpine Summit Vegetation: A Case Study From Kashmir Himalaya
Source: Front Plant Sci. 2020 Apr 24;11:421. doi: 10.3389/fpls.2020.00421 (PMC7194130; doi:10.3389/fpls.2020.00421)
Supplement: Supplementary file 1 [file Data_Sheet_1.pdf]

*Frontiers in Plant Science/Functional Plant Ecology*

## Supplementary material

# Early Evidence of Shifts in Alpine Summit Vegetation: A Case Study from Kashmir Himalaya

*Maroof Hamid*[1*], *Anzar Ahmad Khuroo*[1], *Akhtar Hussain Malik*[1], *Rameez Ahmad*[1], *Chandra Prakash Singh*[2], *Jiri Dolezal*[3,4], *Shiekh Marifatul Haq*[1]

[1]Centre for Biodiversity & Taxonomy, Department of Botany
University of Kashmir, Srinagar, J&K, India

[2]Space Applications Centre, Indian Space Research Organization,
Ahmedabad, India

[3]Institute of Botany, The Czech Academy of Sciences, Zamek,
Pruhonice, Czech Republic

[4]Faculty of Science, Department of Botany, University of South Bohemia,
Ceske Budejovice, Czechia

[*]**Correspondence**

Maroof Hamid

E-mail: hamidmaroofmudasir@gmail.com

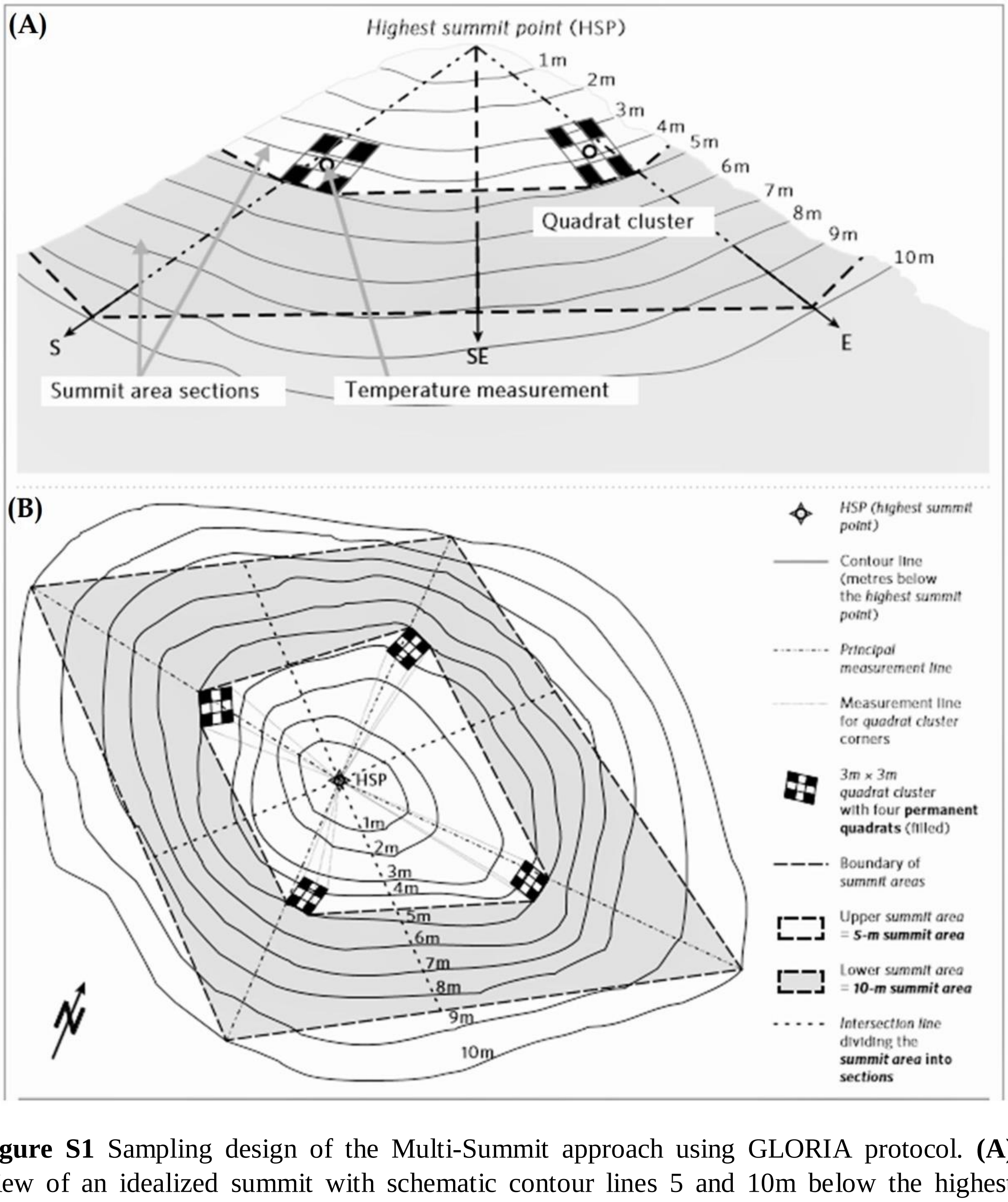


**Figure S1** Sampling design of the Multi-Summit approach using GLORIA protocol. **(A)** View of an idealized summit with schematic contour lines 5 and 10m below the highest summit point. **(B)** Top view of the area sampled (light and dark grey area), showing the four 1 $m^2$ permanent plots (black quadrats), one in each compass direction. Additional explanations can be found in GLORIA field manual (Pauli et al., 2015, Source: The GLORIA field manual—standard multi-summit approach).

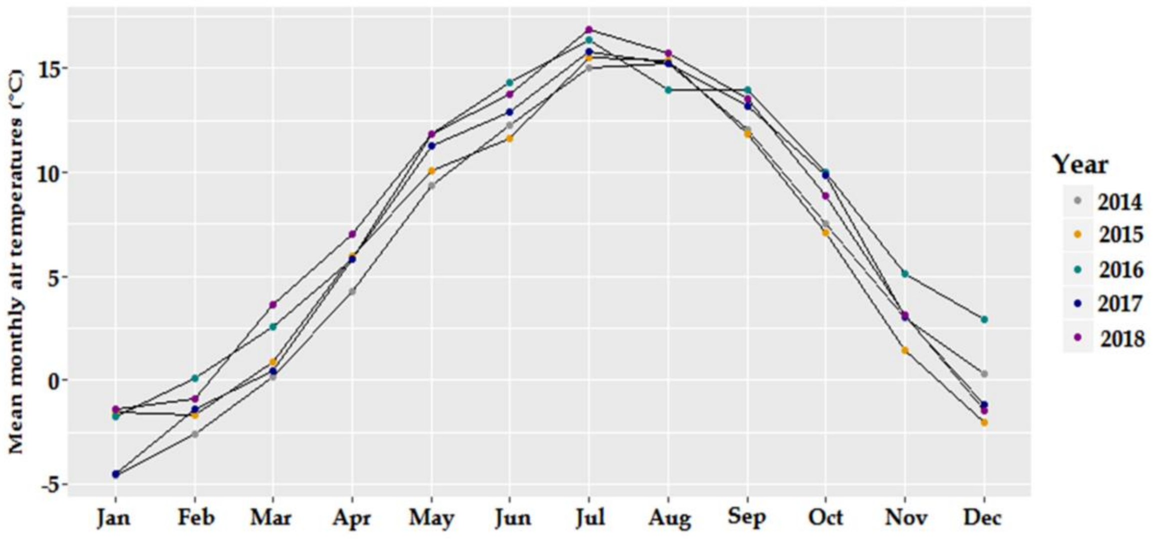


**(A)**

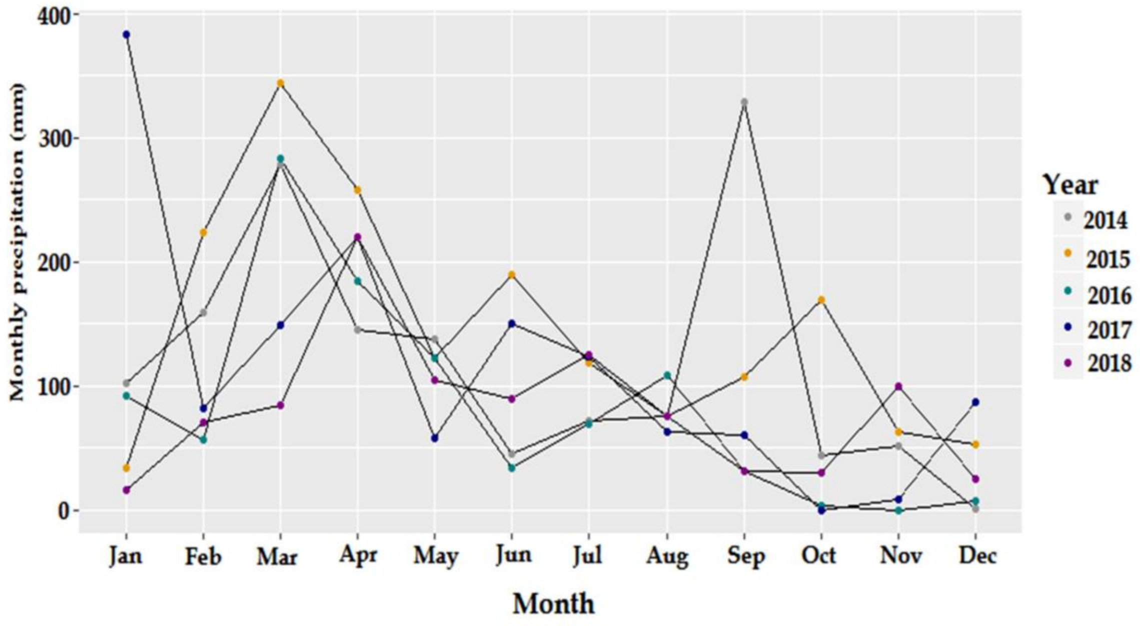


**(B)**

**Figure S2** Climate conditions at the study area between 2014 and 2018. **(A)** Monthly mean air temperatures, **(B)** monthly precipitation.

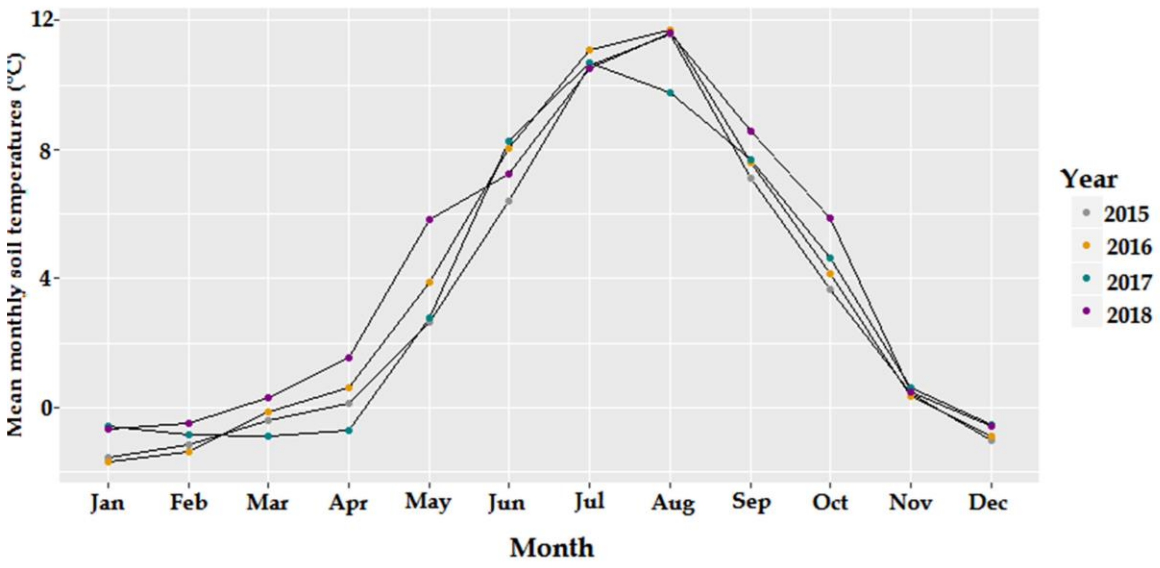


**Figure S3** Monthly mean soil temperatures at the study area between 2015 and 2018.

**Table S1** Description of the five abundance classes or categories, used to visually estimate the species cover percentage within the summit area sections (after Pauli et al., 2015).

| Abundance class | Symbol | Description |
|---|---|---|
| Abundant | a | Very abundant, often forming more or less patchy or dense vegetation layers; species covers more than 50% of the summit area section |
| Common | c | Frequent and widespread occurrence within the section; presence is obvious at the first glance, but it covers less than 50% of the summit area section |
| Scattered | s | Widespread within the section, species can hardly be overlooked, but the presence is not obvious at the first glance; individuals are not necessarily evenly dispersed over the entire summit area section |
| Rare | r | Some individuals at several locations that can hardly be overlooked in a careful observation |
| Very rare | r! | One or a few small individuals |

**Table S2** Description and distribution margins of the elevational ranks applied in the study (after Gottfried et al., 2012).

| Rank | Description | Altitudinal distribution (lower margin / centre / upper margin) |
|---|---|---|
| 1 | Species with a nival distribution centre | nival / nival / nival |
| 2 | Alpine to nival species, not descending to treeline | alpine / alpine / nival |
| 3 | Alpine centred species, not descending to montane belt | treeline / alpine / alpine |
| 4 | Alpine centred species, descending to montane belt or indifferently distributed from the alpine belt to the treeline | montane / alpine / alpine or treeline / indifferent / alpine |
| 5 | Species centred in the treeline ecotone or indifferently distributed from montane to alpine belt | montane / treeline / alpine |
| 6 | Species with a montane distribution centre or indifferently distributed from the montane belt to the treeline | montane / montane / treeline |

**Table S3** Elevational ranks for the vascular plants {based on the species' position on summit, supplemented with local floras (Dhar and Kachroo, 1983; Polunin and Stainton, 1984)}

| **Scientific Name** | **Rank** |
|---|---|
| *Achillea millefolium* L. | 6 |
| *Aconitum heterophyllum* Wall. ex Royle | 4 |
| *Aconitum violaceum* Jacquem. ex Stapf | 3 |
| *Alchemilla trollii* Rothm. | 3 |
| *Anaphalis royleana* DC. | 5 |
| *Androsace rotundifolia* Hardw. | 5 |
| *Anemone obtusiloba* D.Don | 5 |
| *Aquilegia fragrans* Benth | 6 |
| *Arabis tibetica* Hook.f. & Thomson | 4 |
| *Artemisia roxburghiana* Besser | 5 |
| *Aster falconeri* (C.B.Clarke) Hutch. | 5 |
| *Aster thomsonii* C.B.Clarke | 6 |
| *Astragalus grahamianus* Benth. | 6 |
| *Berberis jaeschkeana* C.K.Schneid. | 5 |
| *Bergenia stracheyi* (Hook.f. & Thomson) Engl. | 5 |
| *Betula utilis* D.Don | 6 |
| *Bupleurum candollei* Wall. ex DC. | 5 |
| *Campanula aristata* Wall. | 5 |
| *Carex nubigena* D.Don | 5 |
| *Cassiope fastigiata* (Wall.) D. Don | 2 |
| *Cerastium glomeratum* Thuill. | 5 |
| *Chaerophyllum villosum* Wall. ex DC. | 4 |
| *Chrysopogon gryllus* (L.) Trin. | 5 |
| *Cimicifuga kashmiriana* J.Compton & Hedd. | 6 |
| *Circaea alpina* L. | 5 |
| *Cirsium wallichii* DC. | 6 |

| | |
|---|---|
| *Clinopodium umbrosum* (M.Bieb.) K.Koch | 5 |
| *Codonopsis ovata* Benth. | 5 |
| *Cortia depressa* (D.Don) C.Norman | 5 |
| *Cortusa brotheri* (R.Knuth) Losinsk. | 2 |
| *Corydalis cashmeriana* Royle | 2 |
| *Corydalis diphylla* Wall. | 4 |
| *Corydalis rutifolia* (Sm.) DC. | 4 |
| *Corydalis thyrsiflora* Prain | 5 |
| *Cotoneaster microphyllus* Wall. ex Lindl. | 3 |
| *Cremanthodium decaisnei* C.B.Clarke | 1 |
| *Crepis sancta* (L.) Babc. | 6 |
| *Crucihimalaya himalaica* (Edgew.) Al-Shehbaz, O'Kane & R.A.Price | 4 |
| *Cynoglossum wallichii* G. Don | 6 |
| *Delphinium vestitum* Wall. ex Royle | 4 |
| *Dipsacus inermis* Wall. | 5 |
| *Doronicum roylei* DC. | 5 |
| *Elsholtzia eriostachya* (Benth.) Benth. | 2 |
| *Epilobium parviflorum* Schreb. | 6 |
| *Erigeron multicaulis* Wall. | 5 |
| *Euphorbia wallichii* Hook.f. | 6 |
| *Euphrasia parviflora* Schag. | 5 |
| *Fagopyrum tataricum* (L.) Gaertn. | 3 |
| *Galium aparine* L. | 6 |
| *Gaultheria trichophylla* Royle | 2 |
| *Gentiana carinata* (D. Don) Griseb. | 4 |
| *Geranium nepalense* Sweet | 6 |
| *Geranium pratense* L. | 6 |

| | |
|---|---|
| *Geranium wallichianum* D. Don ex | 6 |
| *Geum elatum* Wall. ex G.Don | 5 |
| *Gypsophila sedifolia* Kurz | 6 |
| *Heracleum candicans* Wall. ex DC | 5 |
| *Hieracium umbellatum* L. | 6 |
| *Impatiens brachycentra* Kar. & Kir. | 3 |
| *Impatiens sulcata* Wall. | 6 |
| *Impatiens thomsonii* Hook.f. | 6 |
| *Inula royleana* DC. | 5 |
| *Jaeschkea canaliculata* Knobl. | 5 |
| *Juniperus squamata* Buch.-Ham. ex D.Don | 4 |
| *Jurinea dolomiaea* Boiss. | 4 |
| *Koeleria pyramidata* (Lam.) P.Beauv. | 5 |
| *Lactuca macrorhiza* (Royle) Hook.f. | 5 |
| *Lamium album* L. | 2 |
| *Leontopodium jacotianum* Beauverd | 5 |
| *Ligularia amplexicaulis* DC. | 1 |
| *Ligularia fischeri* Turcz. | 2 |
| *Lomatogonium caeruleum* (Royle) Harry Sm. ex B.L.Burtt | 5 |
| *Lonicera obovata* Royle ex Hook.f. & Thomson | 3 |
| *Mazus pumilus* (Burm.f.) Steenis | 5 |
| *Meconopsis latifolia* (Prain) Prain | 2 |
| *Melica persica* Kunth | 5 |
| *Morina coulteriana* Royle | 5 |
| *Morina longifolia* Wall. ex DC. | 5 |
| *Myosotis alpestris* F.W.Schmidt | 6 |
| *Myosotis stricta* Link ex Roem. & Schult. | 5 |

| | |
|---|---|
| *Nepeta connata* Royle ex Benth. | 5 |
| *Nepeta laevigata* (D.Don) Hand.-Mazz. | 4 |
| *Nepeta linearis* Royle ex Benth. | 5 |
| *Onosma hispida* Wall. ex G.Don | 6 |
| *Origanum vulgare* L. | 6 |
| *Oxyria digyna* (L.) Hill | 5 |
| *Pedicularis siphonantha* D. Don | 5 |
| *Phleum alpinum* L. | 5 |
| *Phlomoides bracteosa* (Royle ex Benth.) Kamelin & Makhm. | 5 |
| *Picris hieracioides* Sibth. & Sm. | 6 |
| *Pinus wallichiana* A.B. Jacks. | 6 |
| *Plantago himalaica* Pilg. | 5 |
| *Poa angustifolia* L. | 5 |
| *Podophyllum hexandrum* Royle | 6 |
| *Polemonium caeruleum* L. | 5 |
| *Polygonatum multiflorum* (L.) All. | 6 |
| *Polygonum affine* D. Don | 4 |
| *Polygonum amplexicaule* D. Don | 6 |
| *Polypogon fugax* Nees ex Steud. | 5 |
| *Potentilla atrosanguinea* G. Lodd. ex D. Don | 5 |
| *Primula elliptica* Royle | 5 |
| *Primula macrophylla* D. Don | 5 |
| *Primula rosea* Royle | 6 |
| *Pseudomertensia moltkioides* (Royle ex Benth.) Kazmi | 6 |
| *Ranunculus brotherusii* Freyn | 5 |
| *Ranunculus hirtellus* Royle | 6 |
| *Ranunculus palmatifidus* Riedl | 6 |

| | |
|---|---|
| *Rheum webbianum* Royle | 4 |
| *Rhodiola himalensis* (D. Don) S.H. Fu | 6 |
| *Rhododendron anthopogon* D. Don | 3 |
| *Ribes orientale* Desf. | 6 |
| *Rosa webbiana* Wall. ex Royle | 5 |
| *Rumex nepalensis* Spreng. | 6 |
| *Salix denticulata* Andersson | 6 |
| *Salix flabellaris* Andersson | 2 |
| *Salvia hians* Royle ex Benth. | 5 |
| *Saussurea candolleana* Wall. ex C.B.Clarke | 3 |
| *Saussurea costus* (Falc.) Lipsch. | 5 |
| *Saxifraga hirculus* L. | 1 |
| *Saxifraga sibirica* L. | 3 |
| *Scutellaria prostrata* Jacquem. ex Benth. | 5 |
| *Sedum ewersii* Ledeb. | 6 |
| *Senecio chrysanthemoides* DC. | 5 |
| *Sibbaldia cuneata* Edgew. | 5 |
| *Sigesbeckia orientalis* L. | 6 |
| *Silene graminifolia* Otth | 5 |
| *Silene himalayensis* (Rohrb.) Majumdar | 2 |
| *Silene nigrescens* (Edgew.) Majumdar | 3 |
| *Solidago virgaurea* L. | 5 |
| *Stellaria decumbens* Edgew. | 6 |
| *Stipa sibirica* (L.) Lam. | 5 |
| *Swertia petiolata* D. Don | 4 |
| *Tanacetum tibeticum* Hook.f. & Thomson | 4 |
| *Taraxacum officinale* aggr. auct. | 5 |
| *Thalictrum alpinum* L. | 4 |

| | |
|---|---|
| *Themeda anathera* (Nees ex Steud.) Hack. | 5 |
| *Thlaspi cochleariforme* DC. | 4 |
| *Thymus linearis* Benth | 5 |
| *Trifolium pratense* L. | 6 |
| *Trifolium repens* L. | 6 |
| *Valeriana pyrolifolia* Decne | 6 |
| *Verbascum thapsus* L. | 6 |
| *Veronica laxa* Benth. | 5 |
| *Viola biflora* L. | 5 |

**Table S4** One-way ANOVA results for the effect of year on growing degree days (GDD).

| | **df** | **SSR** | **MSR** | **F** | **P** |
|---|---|---|---|---|---|
| Effect of year | 1 | 26 | 26.3 | 0.051 | 0.823 |
| Residuals | 30 | 15509 | 517.0 | | |

**Table S5** A comparison of number of growing degree days (GDD) at summits between August 2014-July 2015 and August 2017-July 2018.

| **Summit** | **Year** | **Aspect** | | | |
|---|---|---|---|---|---|
| | | N | S | E | W |
| **GUL1** | August 2014-July 2015 | 77 | 116 | 109 | 82 |
| | August 2017-July 2018 | 80 | 118 | 112 | 83 |
| **GUL2** | August 2014-July 2015 | 61 | 93 | 98 | 68 |
| | August 2017-July 2018 | 63 | 95 | 98 | 69 |
| **GUL3** | August 2014-July 2015 | 53 | 50 | 78 | 55 |
| | August 2017-July 2018 | 55 | 52 | 80 | 57 |
| **GUL4** | August 2014-July 2015 | 46 | 70 | 67 | 37 |
| | August 2017-July 2018 | 49 | 72 | 68 | 39 |

**Table S6** GUL1 **(A)** One-way ANOVA results for the effect of aspect (nested within summit) on species richness, and **(B)** Tukey multiple comparison test between aspects. Significant differences are indicated as '***'for P < 0.001, '**' P < 0.01, '*' P < 0.05, and '**.**' for P < 0.1.

**(A)**

| | **df** | **SSR** | **MSR** | **F** | **P** |
|---|---|---|---|---|---|
| Effect of aspect (nested within summit) | 3 | 126.5 | 42.17 | 5.988 | 0.0098 ** |
| Residuals | 12 | 84.5 | 7.04 | | |

**(B)**

| **Pair wise comparison** | **Estimate Std.** | **SE** | **t** | **P** |
|---|---|---|---|---|
| N - E | -0.250 | 1.876 | -0.133 | 0.99911 |
| S - E | 5.000 | 1.876 | 2.665 | 0.08401 · |
| W- E | -2.750 | 1.876 | -1.466 | 0.48608 |
| S- N | 5.250 | 1.876 | 2.798 | 0.06705 · |
| W - N | -2.500 | 1.876 | -1.332 | 0.56128 |
| W- S | -7.750 | 1.876 | -4.130 | 0.00656 ** |

**Table S7** GUL2 **(A)** One-way ANOVA results for the effect of aspect (nested within summit) on species richness, and **(B)** Tukey multiple comparison test between aspects. Significant differences are indicated as '***'for P < 0.001, '**' P < 0.01, '*' P < 0.05, and '**.**' for P < 0.1.

**(A)**

| | **df** | **SSR** | **MSR** | **F** | **P** |
|---|---|---|---|---|---|
| Effect of aspect (nested within summit) | 3 | 74.75 | 24.917 | 10.31 | 0.00122 ** |
| Residuals | 12 | 29.00 | 2.417 | | |

**(B)**

| **Pair wise comparison** | **Estimate Std.** | **SE** | **t** | **P** |
|---|---|---|---|---|
| N - E | 3.000 | 1.099 | 2.729 | 0.07520 · |
| S - E | -2.500 | 1.099 | -2.274 | 0.15888 |
| W- E | -2.000 | 1.099 | -1.819 | 0.31152 |
| S- N | -5.500 | 1.099 | -5.003 | 0.00148 ** |
| W - N | -5.000 | 1.099 | -4.549 | 0.00324 ** |
| W- S | 0.500 | 1.099 | 0.455 | 0.96741 |

**Table S8** GUL3 **(A)** One-way ANOVA results for the effect of aspect (nested within summit) on species richness, and **(B)** Tukey multiple comparison test between aspects. Significant differences are indicated as '***'for P < 0.001, '**' P < 0.01, '*' P < 0.05, and '**.**' for P < 0.1.

**(A)**

| | **df** | **SSR** | **MSR** | **F** | **P** |
|---|---|---|---|---|---|
| Effect of aspect (nested within summit) | 3 | 185.19 | 61.73 | 14.6 | 0.000262 *** |
| Residuals | 12 | 50.75 | 4.23 | | |

**(B)**

| **Pair wise comparison** | **Estimate Std.** | **SE** | **t** | **P** |
|---|---|---|---|---|
| N - E | 2.500 | 1.454 | 1.719 | 0.35614 |
| S - E | -2.750 | 1.454 | -1.891 | 0.28198 |
| W- E | 6.500 | 1.454 | 4.470 | 0.00377 ** |
| S- N | -5.250 | 1.454 | -3.610 | 0.01630 * |
| W - N | 4.000 | 1.454 | 2.751 | 0.07250 · |
| W- S | 9.250 | 1.454 | 6.361 | < 0.001 *** |

**Table S9** GUL4 **(A)** One-way ANOVA results for the effect of aspect (nested within summit) on species richness, and **(B)** Tukey multiple comparison test between aspects. Significant differences are indicated as '***'for P < 0.001, '**' P < 0.01, '*' P < 0.05, and '**.**' for P < 0.1.

**(A)**

| | **df** | **SSR** | **MSR** | **F** | **P** |
|---|---|---|---|---|---|
| Effect of aspect (nested within summit) | 3 | 153.2 | 51.08 | 29.9 | < 0.001*** |
| Residuals | 12 | 20.5 | 1.71 | | |

**(B)**

| **Pair wise comparison** | **Estimate Std.** | **SE** | **t** | **P** |
|---|---|---|---|---|
| N - E | -0.7500 | 0.9242 | -0.812 | 0.8479 |
| S - E | 1.7500 | 0.9242 | 1.894 | 0.2810 |
| W- E | -6.5000 | 0.9242 | -7.033 | <0.001 *** |
| S- N | 2.5000 | 0.9242 | 2.705 | 0.0785 · |
| W - N | -5.7500 | 0.9242 | -6.222 | <0.001 *** |
| W- S | -8.2500 | 0.9242 | -8.927 | <0.001 *** |

**Table S10 (A)** One-way ANOVA results for the effect of summit on species richness, and **(B)** Tukey multiple comparison test between summits. Significant differences are indicated as '***'for P < 0.001, '**' P < 0.01, '*' P < 0.05, and '**.**' for P < 0.1.

**(A)**

| **Summit** | **df** | **SSR** | **MSR** | **F** | **P** |
|---|---|---|---|---|---|
| Effect of summit | 3 | 947.6 | 315.88 | 28.62 | < 0.001*** |
| Residuals | 124 | 1368.7 | 11.04 | | |

**(B)**

| **Pair wise comparison** | **Estimate Std.** | **SE** | **t** | **P** |
|---|---|---|---|---|
| GUL2-GUL1 | -3.1563 | 0.8306 | -3.800 | 0.00131 ** |
| GUL3-GUL1 | -7.5938 | 0.8306 | -9.143 | < 0.001 *** |
| GUL4-GUL1 | -4.4062 | 0.8306 | -5.305 | < 0.001 *** |
| GUL3-GUL2 | -4.4375 | 0.8306 | -5.343 | < 0.001 *** |
| GUL4-GUL2 | -1.2500 | 0.8306 | -1.505 | 0.03761 * |
| GUL4-GUL3 | 3.1875 | 0.8306 | 3.838 | 0.00114 ** |

**Table S11** Two-way ANOVA results for the effect of summit and year of sampling on species richness. Significant differences are indicated as ‘***’for P < 0.001, ‘**’ P < 0.01, ‘*’ P < 0.05, and ‘**.**’ for P < 0.1.

| | **df** | **SSR** | **MSR** | **F** | **P** |
|---|---|---|---|---|---|
| Effect of summit | 3 | 947.6 | 315.88 | 28.62 | < 0.001*** |
| Effect of year | 1 | 59.1 | 59.13 | 5.424 | 0.0215 * |
| Interaction effect between summit and year | 3 | 50.2 | 12.40 | 3.37 | 0.0404 * |
| Residuals | 120 | 1308.3 | 10.90 | | |

**Table S12** Two-way ANOVA results for the effect of summit and aspect on soil temperature. Significant differences are indicated as ‘***’for P < 0.001, ‘**’ P < 0.01, ‘*’ P < 0.05, and ‘**.**’ for P < 0.1

| | **df** | **SSR** | **MSR** | **F** | **P** |
|---|---|---|---|---|---|
| Effect of summit | 3 | 53.98 | 17.995 | 7662.5 | < 0.001*** |
| Effect of aspect | 3 | 24.51 | 8.171 | 3479..3 | < 0.001*** |
| Interaction effect between summit and aspect | 9 | 14.01 | 1.557 | 662.8 | < 0.001*** |
| Residuals | 48 | 0.11 | 0.002 | | |
